# Perspectives on Movement and Eating Behaviours in Brazilian Elderly: An Analysis of Clusters Associated with Disease Outcomes

**DOI:** 10.14336/AD.2022.0131

**Published:** 2022-10-01

**Authors:** Mabliny Thuany, Douglas Vieira, Anderson Santana Santos, Ewa Malchrowicz-Mosko, Thayse Natacha Gomes

**Affiliations:** ^1^CIFI2D, Faculty of Sports, University of Porto, 4200-450, Porto, Portugal.; ^2^Post-Graduation Program of Physical Education, Federal University of Sergipe - São Cristóvão-SE, Brazil.; ^3^Faculty of Sport Sciences, Poznan University of Physical Education, Poznan, Poland.; ^4^Department of Physical Education, Federal University of Sergipe, São Cristóvão-SE, Brazil.

**Keywords:** aging, health, movement behaviour, eating habits, latent class analysis

## Abstract

Aging is a biological process, which is usually associated with health-related problems, which are related to some behaviours, such as those related to movement and eating habits. So, the purpose of the present study was to identify the clustering of behavioural and eating habits related to non-communicable disease in Brazilian elderly, and to estimate the association of these profiles with overweight/obesity, hypertension, and diabetes. This is a cross-sectional based-population study, which sample comes from the VIGITEL 2019 survey. The sample comprised 23,327 subjects (16,295 women), mean age of 71 years. Sociodemographic and anthropometric data (i.e., age, sex, body weight, and body height), health-related information (i.e., eating habits, physical activity and sedentary behaviour, and alcohol consumption), health status and morbidity (diabetes and hypertension) were self-reported. Latent Classes Analysis, and binary logistic regression were performed, considering p<0.05. Results showed that two different classes were identified. Those called as “TV viewer, but no unhealthy diet” presented more chances to have hypertension (OR: 1.213; 95%CI: 1.064-1.382) and diabetes (OR: 1.365; 95%CI: 1.157-1.610), when compared to their peers called as “healthy diet and active”. Age, educational level, and sex were associated with hypertension and diabetes. In conclusion, a better health clustered-behaviour was associated with better disease outcomes in Brazilian elderly population.

Aging is a biological process, which is usually associated with health-related problems [1]. Due to improvements in quality of life and health care, increases in life expectancy has been observed worldwide, and data from the World Health Organization estimates that, in 2050, the world’s population over 60 years will reach 22%, outnumbering children younger than 5 years (www.who.int/news-room/fact-sheets/detail/ageing-and-health). In addition, about 80% of these subjects will be living in low- and middle-income countries, highlighting the huge number of challenges that health system will face (www.who.int/news-room/fact-sheets/detail/ageing-and-health).

From the health-related problems associated with aging, the non-communicable diseases are the most common, such as cardiovascular disease, hypertension, diabetes, obesity, cancer, dementia, and so on. Further, evidence suggests that most of the elderly tend to have more than one comorbidities, increasing the risk of disability and mortality [2], and also increasing the public health costs.

Given that, it is of relevance to promote a more health and active lifestyle among this group, since is knowing that the adoption of some behaviours, such as adequate levels of physical activity, and a diet behaviour based on *in natura* or low processed foods, can reduce the risks of development of non-communicable diseases, preventing physical and cognitive decline during the aging process [3, 4]. Physical activity proved to be able to assist older adults in the context of healthy aging and better quality of life [5, 6]. However, physical inactivity has been pointed as one of the main public health problems worldwide, with estimates that, globally, ≈5.3 million deaths can be attributed to comorbidities associated with physical inactivity [7]. Moreover, evidence shows that the adoption of sedentary behaviour is directly linked to deaths from cardiovascular diseases [8].

In addition, the increase in ultra-processed food consumption is related to increases in non-communicable disease and also their related-risk factors, such as obesity [4]. Especially among elderly, available information report that only ≈40% of them properly consume the recommended portions of fruits and vegetables [9], but discrepancies in the prevalence can be observed when taking into account socioeconomic disparities [10].

At the Brazilian context, available data indicates an increment of 29.5% in elderly population, and most of them do not reach the physical activity recommendations (69.1%). While regarding food consumption, about only a quarter of subjects aged ≥65 years comply with the daily recommendation of fruit and vegetables [11]. In addition, more than half of people aged ≥65 years has hypertension [12, 13] or is classified as overweight (www.pns.icict.fiocruz.br/painel-de-indicadores-mobile-desktop/).

So, since non-communicable diseases are the main cause of death, especially among older adults, in both global and Brazilian context (www.healthdata.org/brazil), and given the fact that the adoption of healthy lifestyle can improve the quality of life and reduce the risk of development of these diseases and/or their risk factors, allowing the reduction of public health system costs, it seems of relevance understanding the aggregation of behaviours that can increase the chances of development of unhealthy outcomes, which can provide relevant information to be used in the development of public health approaches. Thus, this study aimed to identify the clustering of movement behavioural and diet habits related to non-communicable disease in Brazilian elderly, and to estimate the association of these profiles with overweight/obesity, hypertension, and diabetes.

## MATERIALS AND METHODS

### Sample

This is a cross-sectional based-population study, which sample comes from the VIGITEL 2019 survey (approved by the National Commission for Ethics in Research with Human Beings of the Ministry of Health - CONEP 355590). This is a telephone survey, which is carried out annually since 2006, sampling adults aged ≥18 years, living in all the 26 Brazilian capital cities and in the Federal District, in homes with at least one fixed phone line.

For the present study, only those aged ≥60 years, and with no missing data for the variables used in the analysis, were considered. Thus, the sample was comprised for 23,327 subjects (16,295 women, 7,032 men), aged between 60 and 106 years (mean age, 71 years). The data collection was performed through a phone survey, and participants were asked to answer a questionnaire, providing information regarding sociodemographic data, health-related information (eating habits, physical activity and sedentary behaviour, smoking and alcohol consumption), anthropometric data (height and weight), self-reported health status and morbidity. More details regarding sampling selection and data collection can be obtained in published works [11].

### Demographic and biological variables

Age, sex, body weight, and body height were self-reported. Body mass index was computed, based on standardized formula [body weight(kg)/body height(m)^2^], and subjects were classified as normal weight or overweight/obese, according to WHO cut points [14, 15].

School level was determined based on the years of official formal education that participants reported having attended.

### Physical activity and sedentary behaviour

Information regarding physical activity were informed, and computed, in different domains (leisure, commuting, work, and domestic chores). For the present study, information regarding physical activity in leisure time, commuting, and work was used.

For leisure physical activity, participants indicated their involvement in physical exercise or sports, during their leisure time, in the last three months (modality, frequency/week, and session duration). Participants were also asked to inform about their commuting to work/school/faculty (if active or inactive, through motorized transport), and the time they spent in this kind of activity. Further, regarding work, subjects were asked to inform if they got involved in physical activities during their labour, and the time spent on it. Subjects were classified as “active”, if they reached at least 150min of moderate to vigorous physical activity and/or at least 75min of vigorous physical activity, per week, taking into account the sum of time spent in physical activities of these intensities in the three mentioned domains (those who did not reach the recommendation, were classified as “inactive”).

TV time was used as sedentary behaviour. Participants reported the mean time spent watching tv/day. Based on answers provided, they were classified as “<3h/day TV” or “≥3h/day TV”.

### Food consumption and alcohol use

Participants reported the frequency of food consumption related to the development of non-communicable diseases, in the day before the interview. Foods were categorized in two groups, namely “ultra-processed foods” and “in natura/unprocessed or minimally processed foods”, highlighting the healthy and unhealthy diet. Thus, for “in natura/unprocessed or minimally processed foods”, if subjects reported to had consumed five or more portions of foods from this groups, they were classified as achieving the guidelines for healthy diet; regarding “ultra-processed foods”, if they reported the consumption of five or more portion of foods from this groups, they were classified as not achieving the guidelines for ultra-processed food consumption.

Regarding the consumption of fruits and vegetables, subjects indicated the frequency they eat these foods, in a typical week. Those who reported to consume them in, at least, five days/week were classified as achieving the recommendations.

For alcohol consumption, participants were classified as having an abusive consumption if they reported to consume at least five (men) or four (women) alcohol drinks in a day, in the 30 days before the interview.

### Non-communicable diseases

Participants answered if they were previously diagnosed as having diabetes and hypertension. Positive answers indicated the existence of the disease.

### Statistical analysis

Descriptive data were presented as mean (standard deviation) and frequency (%). To identify groups as subjects according to their behavioural and nutritional habits, the Latent Classes Analysis was performed, in the Mplus software, taking into account the following variables: “consumption of fruits/vegetables” (<5 days/week or ≥ 5 days/week), “in natura/unprocessed or minimally processed foods” (<5 portions/day or ≥ 5 portions/day), “ultra-processed foods” (<5 portions/day or ≥ 5 portions/day), “TV time” (<3h/day or ≥ 3h/day), “abusive alcohol consumption” (no or yes), “physical activity” (inactive or active), and the first class, for all the variables, was used as reference.

Binary logistic regression analysis was computed to estimate variables related with the chances of subjects having a non-communicable chronic disease (diabetes, hypertension, overweight/obesity), including, as predictors, the classes derived from the latent class analysis, in addition to sex, age, and school-level. The analysis considered the survey sampling complex plan (SCP), as suggested (http://svs.aids.gov.br/download/Vigitel/Orientacoes-sobre-o-uso-das-bases-de-dados.pdf), and it was performed in the SPSS software v. 26, with a significance level at 5%.

## Brazilian elderly lifestyle and health outcomes

**Figure 1.**
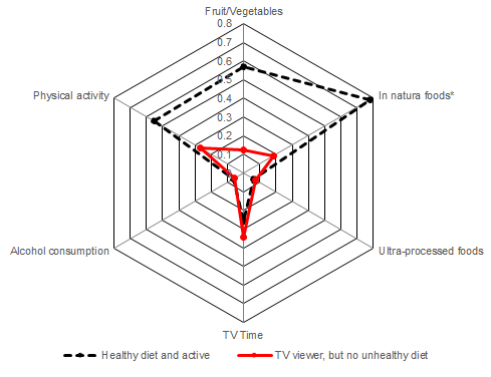
**Profiles derived from the latent class analysis**. Legend: *in natura/unprocessed or minimally processed foods

The latent classes analysis showed the existence of two different classes, based on nutritional and behavioural habits of the participants, which were called as “healthy diet and actives” and “TV viewer but no unhealthy diet”. As illustrated in Figure 1, subjects from the “healthy diet and actives” class showed more probabilities to achieve the guidelines regarding the consumption of fruits/vegetables and in natura/unprocessed or minimally processed foods, and also the physical activity guidelines; on the other hand, the “TV viewer but no unhealthy diet” class subjects showed more probabilities to spend 3 or more hours/day watching TV, but presented low probabilities to unhealthy diet habits or achieve the healthy diet guidelines.

Despite the differences between classes regarding subjects’ involvement in physical activities, both of them did not present a high consumption of ultra-processed food. A review conducted by Marino et al highlights that notwithstanding the consumption of ultra-processed foods be observed in all age groups, a decreasing of its frequency consumption is observed with increasing age, where the highest level of ultra-processed food intake found among children and adolescents, while the lowest frequency being observed among the oldest subjects [16], and this can explain the fact that both groups did not present a high frequency of ultra-processed food intake.

On the other hand, differences in movement behaviour were observed between classes, where one of the classes presented a high frequency of subjects physically active, while the other presented a high frequency of subjects who spend more time on TV. It is well knowing that physical activity decreases with increasing age, meaning that the frequency of inactivity tends to be higher in older people, when compared to the youngest ones [17]. From the set of possible variables related to it, can be mentioned changes in physiological/biological characteristics, economic and social aspects, as well as environmental constraints that can act as barriers to elderly physical activity involvement.

Variables distribution, according to the two classes, is presented on Table 1. Mean age and sex distribution are similar for both classes, as well as the frequency of subjects with abusive alcohol consumption. More than half percent of subjects from “TV viewer, but no unhealthy diet” class just completed until the elementary school, and almost a quarter of them reported to be diagnosed as diabetics. Almost 60% of those from “healthy diet and actives” class achieved the physical activity guidelines, and more than 80% of them consumed fruits/vegetables and in natura/unprocessed or minimally processed foods as recommended.

Table 2 shows results for the logistic regression analysis, for the chances of subjects be overweight/obese, having hypertension or diabetes, respectively. For over-weight/obesity, only age showed to be a significant predictor, highlighting that increasing age, decreases the odds of subjects having risk regarding to excess weight (OR: 0.979, 95%CI: 0.972-0.986). However, age was a significant predictor for hypertension, increasing the chances of subjects present elevated blood pressure with increasing their ages (OR: 1.016, 95%CI: 1.009-10.24). Aging process is closely related to the development of chronic disease, such as those investigated in the present study [18]. In addition, excess weight is one risk factor for hypertension [19]. So, results found ca be related to the fact that, maybe, older subjects tend to better control their weight, with the purpose to minimize the risk of having hypertension, as well as those already diagnosed as hypertensive may control their weight with the purpose to reduce the health risks associated with both conditions. It is important also to point out that increasing age leads to reduction in body mass, which can be related to decreases in body weight, regardless of weight control [20, 21].

In addition, school-level was also a significant predictor for both, hypertension and diabetes, meaning that higher the subjects’ school-level, lower the odds of being diagnosed with these diseases. This can be due to the fact that high educational level seems to be associated with the adoption of healthier eating patterns, a behaviour that is directly related to the prevention of cardiovascular disease [22]. Furthermore, education level is related to some social issues, such as better economic condition, living and working in healthier environments, having access to health services and physical activity practices possibilities, which are factors also related to the development/prevention of cardiovascular diseases, such as diabetes and hypertension [23].

**Table 1:** Descriptive statistics [mean (standard error) or frequency] for variables included in the study, by class.

Variables	Healthy diet and active (n=7,086)	TV viewer but no unhealthy diet (n=16,241)
**Age (years)**	68.03 (0.17)	69.68 (0.13)
**Sex**		
***Female***	61.4%	61.2%
***Male***	38.6%	38.8%
**School level**		
***0-8 years***	45.6%	66.4%
***9-11 years***	24.8%	21.2%
***≥ 12 years***	29.6%	12.5%
**Fruits/Vegetables (yes)**	82.0%	28.2%
In natura foods (yes)*	83.5%	14.6%
**Ultra-processed foods (yes)**	7.8%	9.0%
**Abusive alcohol consumption (yes)**	6.5%	5.9%
**≥3h/day TV (yes)**	27.1%	33.8%
**Physical activity (active)**	59.4%	14.1%
**Overweight/Obese**	59.6%	62.2%
**Hypertension (yes)**	49.8%	57.6%
**Diabetes (yes)**	16.8%	23.6%

Legend: mean (standard error),* In natura/unprocessed or minimally processed foods

Furthermore, while men were more prone to have diabetes (OR: 1.202, 95%CI: 1.037-1.394), regarding hypertension they had lower chances, compared to women (OR: 0.809, 95%CI: 0.714-0.917). The fact that women are more likely to develop hypertension may be related to hormonal issues [24]. During their reproductive period they usually present lower blood pressure than men; but after the menopause, with the reduction of estrogen, which is a cardioprotective sexual hormone, blood pressure among women tends to increase, and this fact can be related to the higher odds of women be hypertensive compared to men in the age group studied [25, 26]. In addition, some behaviours, such as low physical activity levels (in general, women are less active then men), and also diet habits, can contribute, in association with the mentioned physiological changes, to this scenario [27, 28]. On the other hand, men have more chances to present diabetes, and this result is in accordance with previous study [29]. This can be related to lifestyle, where, in general, men tend to have higher caloric intake and alcohol consumption than women, which are two risk behaviours related to diabetes, in association with the fact that physical activity (which is a protective factor against most chronic disease) tend to decline with aging process [30].

The “TV viewer, but no unhealthy diet” class was a significant predictor for hypertension (OR: 1.213; 95%CI: 1.064-1.382) and diabetes (OR: 1.365; 95%CI: 1.157-1.610), and subjects from this class were more prone to present the mentioned disease than their pairs from the “healthy diet and active” class. It is important to note that none of the groups presented a high ultra-processed food consumption, but subjects from the class “healthy diet and active” were more active and showed to have a higher intake of fruit/vegetables and in natura/unprocessed or minimally processed food. It is well knowing that physical activity plays a relevant role in the prevention of chronic diseases risk factors [31, 32], and findings also reveal a strong correlation between physical activity and cardiorespiratory fitness, the nervous system, psycho-motor skills, vascular aging, mood, cognition, and the overall quality of life as a result of a regular non-pharmacological treatment [33]. In addition, neuroscience provides evidence that in the older people exercises enhance plasticity of the brain networks [3]. Furthermore, studies also point the positive role of a healthy diet in the prevention of these diseases [34]. So, these results may highlight the relevance of the adoption of an active lifestyle, in association with a diet with high intake of in natura/unprocessed or minimally processed food, in the prevention for the development of chronic disease, especially those related to the cardiovascular system.

**Table 2:** Regression logistic analysis of associated factor for overweight/obesity, hypertension and diabetes.

	Overweight/obesity	Hypertension	Diabetes
**Predictor**	OR (95%CI)	OR (95%CI)	OR (95%CI)
**Sex (male)**	0.98 (0.871 - 1.123)	0.80(0.71 - 0.91)	1.20(1.03 - 1.39)
**Age (years)**	0.97 (0.972 - 0.986)	1.01(1.00 - 10.24)	1.00(0.99 - 1.01)
***School level***			
**0-8 years**	1	1	1
**9-11 years**	0.93(0.81 - 1.06)	0.81(0.71 - 0.93)	0.79(0.67 - 0.93)
**≥12 years**	0.90(0.77 - 1.04)	0.58(0.50 - 0.67)	0.51 (0.42 - 0.63)
**TV viewer, but no unhealthy diet**	1.13(0.99 - 1.29)	1.21(1.06 - 1.38)	1.36(1.15 - 1.61)

Abbreviations: OR, odds ratio. 95%CI, 95% confidence interval, bold, significant association.

In summary, regardless of movement behaviours (i.e., physical activity and TV time), Brazilian elderly population did not present a high consumption of ultra-processed food. For the two classes identified, those called as “TV viewer, but no unhealthy diet” were more prone to have hypertension and diabetes. In addition, increasing educational level, decreases the chances of having hypertension or diabetes. Results may highlight the relevance of physical activity, in this group, as a protective factor against the development of chronic disease. Thus, programs aiming to promote physical activity within this age-group could be of relevance to promote a better health-status among them, and also could positively impact the public health costs. Some limitations can be pointed from this study. Firstly, we can highlight that self-reported information is prone to bias and may does not reveal the real health status of the participants. Secondly, the lack of information regarding the environmental characteristics that can be related to subjects’ physical activity and food consumption can impair the generalization of the results, especially when focusing to understand between-states differences. On the other hand, considering the increment of life expectance among Brazilian population, we highlight that information can be used for the development of public policy programs, especially those able to be executed in the public health system, aiming to increment physical activity levels among this group, considering age, sex and educational level. Furthermore, longitudinal studies could provide information regarding the long-term relationship between movement behaviours and food consumption with the development of chronic diseases.

## Perspectives

As found, elderly population who spend more time in sedentary behaviour and have an inadequate diet are more likely to present negative health outcomes. So, given the positive trend regarding the number of the Brazilian elderly population alongside the recent years (www.ibge.gov.br/estatisticas/sociais/trabalho/17270-pnad-continua.html?edicao=18264&t=o-que-e; www.ibge.gov.br/apps/populacao/projecao/index.html), similar to what is observed in others mid-high-income countries around the world (https://population.un.org/wpp/Download/Standard/Population/), results of the present study can be useful to the development of evidence-based programs.

In addition, professional guidance regarding the adoption of healthy habits, may lead to the increment in the compliance of health diet and behaviours/movements guidelines [35]. Thus, the development and/or improvement of strategies aiming to health promotion among elderly population can contribute to the adherence of healthy behaviours, such as physical activity and adequate nutrition, reducing the chances of the development of the studied chronic diseases.
